# Cardiovascular risk management in patients using antipsychotics: it is time to take action

**DOI:** 10.1186/s12916-020-01811-7

**Published:** 2020-11-02

**Authors:** Erik W. M. A. Bischoff, Kirsti M. Jakobs, Willem J. J. Assendelft

**Affiliations:** Department of Primary and Community Care, Radboud University Medical Center, P.O. Box 9101, 6500 HB Nijmegen, The Netherlands

**Keywords:** Cardiovascular risk management, Antipsychotic drugs, Primary care

## Background

The study from Lai and colleagues, recently published in *BMC Medicine*, provides interesting new insights on sex-related associations between antipsychotic use and acute ischemic heart disease [1]. The authors demonstrated that antipsychotic use was associated with a 32% increased hazard rate of acute ischemic heart disease (IHD) among women (95% CI 1.05–1.67), but not among men. In their Hong Kong primary care population, almost 2% were prescribed antipsychotic drugs.

The use of antipsychotic medications is increasing worldwide. Antipsychotics are indicated for the treatment of severe mental illness (SMI), including psychotic and bipolar disorder. Remarkably, a large proportion of patients on antipsychotics do not have a diagnosis of SMI. This off-label use can add up to 60% of antipsychotic prescriptions, particularly for atypical antipsychotic drugs such as olanzapine [2]. Reasons for off-label prescriptions are anxiety, depression, dementia, sleep, and personality disorders [2]. As a result of such increased use, long-term side effects of antipsychotic drugs may increase the burden on patients and healthcare services globally. Long-term use may affect metabolic pathways, thereby causing weight gain, glucose intolerance, dyslipidemia, and cardiac toxicity, resulting in an increased risk of diabetes, cardiovascular disease, and mortality [3]. Current guidelines on cardiovascular risk management, such as those from the National Institute for Health and Care Excellence (NICE) [4], are particularly relevant for cardiometabolic risk in patients on antipsychotic drugs, specifically atypical antipsychotics. However, risk management in patients on antipsychotics is often performed poorly [5]. A recent study showed screening rates in less than 10% of Dutch primary care patients using antipsychotics [6]. What do the study results of Lai and colleagues add to current clinical guidelines? And do their results support further improvement of cardiovascular risk management implementation?

## Variations in side effects and the influence of patient factors

Lai and colleagues performed a retrospective study using primary care data of over one million patients. A retrospective design, however, is unsuitable to prove causality. Important known intermediate variables, like cholesterol and blood glucose level, and an unhealthy lifestyle, were not taken into account. Therefore, confounding by indication cannot be ruled out. The same problem occurs with dosage, duration of the use, and the underlying diagnosis.

Research increasingly suggests that patients on antipsychotic drugs are in high need of cardiometabolic risk screening [3]. However, the risk of an individual patient appears to depend on many personal circumstances and variables, which makes estimation of the cardiometabolic risk difficult. A recent meta-analysis on the efficacy and tolerability of 32 oral antipsychotics showed large variations among antipsychotic drugs in metabolic side effects and that specific patient factors, such as age, sex, and ethnicity, may increase the risk of metabolic dysregulation [7]. Despite the limitations in the study of Lai and colleagues, their results also suggest variations in side effects and the influence of patient factors. The association found between antipsychotic drugs and ischemic heart disease in women weakened and became non-significant after omitting haloperidol, a typical antipsychotic, from the analyses. Variations in side effects and patient-related factors should be taken into account by healthcare professionals when providing cardiovascular risk management for individuals using antipsychotics.

## Cardiovascular risk management

An increased cardiovascular risk in patients taking antipsychotic medications can be better treated in a primary care setting, with lifestyle counseling as well as pharmacological interventions, not differently from managing cardiovascular risk in other patient groups [4]. Besides, primary care has the opportunity to reach a broad population, including patients with mental disorders in a stable phase using antipsychotics and as a consequence not under regular specialist care. However, family physicians may be insufficiently aware of specific antipsychotic side effects, interactions, and relevant patient factors, which may hinder the required personalization of cardiovascular risk management. For instance, dose reduction and switching to an antipsychotic drug with a better metabolic profile are promising strategies to lower cardiometabolic risk. Moreover, barriers in access and communication between family physicians and patients using antipsychotic drugs may further complicate implementation of cardiovascular risk screening and treatment. Healthcare professionals are inconsistent in their approach, and sometimes have negative perceptions towards patients with SMI, for instance regarding smoking cessation. Simultaneously, patients’ access to primary care for the target group at issue is often hindered by limited help-seeking behavior, psychological barriers, and poor understanding of preventing physical illness. The complexities regarding implementation of cardiovascular risk management in patients using antipsychotics require well-designed complex interventions, in which family physicians closely collaborate with patients, psychiatrists, and other disciplines. In this context, consultation liaisons and collaborative care models are options to consider. In consultation liaisons, family physicians maintain the central role in the delivery of care with mental health specialists providing consultative support. The collaborative care model is a broader, more systematic approach that involves the integration of care managers and consultant psychiatrists, controlled by the family physician. Both models have shown positive results in the primary care for people with mental disorders [8], but not on the lowering of cardiovascular risk in this specific population [9]. While developing interventions, healthcare professionals and researchers should consider that the causal chain between a risk lowering intervention and a desired outcome is complex and easily disrupted. The guideline for the development and evaluation of complex interventions of the Medical Research Council may be supportive [10].

## Time to take action

Given the complex nature of causal factors (indications for prescription), the unknown impact of the various intermediate factors (pathophysiological and biochemical parameters; lifestyle factors), and the unknown effectiveness of the required complex interventions, we argue that the study by Lai and colleagues is exploratory in nature and should be applied carefully in clinical practice. However, the evidence about the considerable risks of antipsychotics is convincing enough to take action and effectively implement cardiovascular risk management interventions, based on collaborative care models and consultation liaisons. Future studies should focus on the development and evaluation of these complex interventions, and include closely monitoring the intermediate variables, to further untangle the roles of antipsychotic drugs in increasing cardiovascular risk.

## Data Availability

Not applicable.
